# Predicting physical activity change in cancer survivors: an application of the Health Action Process Approach

**DOI:** 10.1007/s11764-021-01107-6

**Published:** 2021-09-13

**Authors:** Sarah J. Hardcastle, Chloe Maxwell-Smith, Martin S. Hagger

**Affiliations:** 1 School of Health and Human Performance, Dublin City University, Dublin, Ireland; 2 Institute for Health Research, The University of Notre Dame Australia, Fremantle, WA Australia; 3 School of Psychology, Curtin University, Perth, WA Australia; 4 Department of Psychological Sciences and Health Sciences Research Institute, University of California, Merced, CA USA; 5 Faculty of Sport and Health Sciences, University of Jyväskylä, Jyväskylä, Finland

**Keywords:** Action planning, Behavior change, Cancer survivors, Exercise, Theory, Oncology

## Abstract

**Purpose:**

Previous research has not examined the utility of the Health Action Process Approach (HAPA) to predict physical activity (PA) change in cancer survivors. The aim of the study was to investigate the efficacy of a HAPA-based model in predicting temporal change in moderate-to-vigorous physical activity (MVPA) in cancer survivors.

**Methods:**

Participants enrolled in the Wearable Activity Technology and Action Planning (WATAAP) trial completed validated questionnaires (*n* = 64) to assess HAPA constructs (action and maintenance self-efficacy, outcome expectancies, action planning, risk perceptions, and intention) and wore an ActiGraph to measure PA at baseline, 12 weeks, and 24 weeks later. Data were analyzed using variance-based structural equation modeling with residualized change scores for model variables.

**Results:**

Consistent with predictions, changes in action self-efficacy (*β* = 0.490, *p* < 0.001, ES = 0.258) and risk perceptions (*β* = 0.312, *p* = 0.003, ES = 0.099) were statistically significant predictors of intention change over time. Changes in intention (*β* = 0.217, *p* = 0.029, ES = 0.040) and action planning (*β* = 0.234, *p* = 0.068, ES = 0.068) predicted changes in MVPA. Overall, the model accounted for significant variance in intention (*R*^2^ = 0.380) and MVPA (*R*^2^ = 0.228) change.

**Conclusions:**

Changes in intention and action planning were important correlates of MVPA change over 24 weeks. Further, changes in action self-efficacy and risk perceptions predicted changes in intention. Implications for cancer survivors: interventions that foster risk perceptions and self-efficacy, strengthen intentions, and promote action planning may be effective in promoting sustained PA change in cancer survivors.

**Supplementary Information:**

The online version contains supplementary material available at 10.1007/s11764-021-01107-6.

## Introduction

Improved detection and treatment have led to increased cancer survival [1, 2] with two-thirds of patients living beyond 5 years of diagnosis [3]. Interventions that focus on reducing comorbidities, cardiovascular disease (CVD), and cancer recurrence, and improving quality of life have been shown to facilitate healthy survivorship trajectories [4]. The promotion of physical activity (PA) is a promising strategy to reduce the risks of CVD and cancer recurrence. Physically active survivors have lower CVD-related morbidity [5], lower recurrence risk, and improved survival compared to those who are insufficiently active [6]. The American Cancer Society recommends that survivors participate in a minimum of 150 min of moderate-intensity PA per week [7]. However, less than 30% of survivors meet these guidelines [7].

There has therefore been a proliferation in interventions to increase PA in survivors and research endeavors to better understand the social-psychological factors that influence PA in this group [8]. Understanding the psychosocial factors underpinning PA behavior change is important to identify potentially modifiable factors that could be targeted in interventions to increase PA. The application of theories in this endeavor is important because theories facilitate understanding of the determinants of behavior and the associated mechanisms by which those determinants relate to behavior [9, 10].

A prominent theoretical model that has been adopted to identify the determinants of PA is the Health Action Process Approach (HAPA). The HAPA is a social cognition model that identifies the belief-based determinants of intentions and behavior and the processes involved. The HAPA also incorporates additional constructs aimed at resolving the frequently reported intention-behavior “gap” [11]. Specifically, two phases required for behavior change are proposed: motivational and volitional phases [12]. The motivational phase encompasses constructs involved in intention formation, including outcome expectations, confidence to engage in PA, known as action self-efficacy, and risk perceptions [12]. The volitional phase encompasses constructs involved in the enactment of behavior after an intention has been formed to bridge the intention-behavior gap. Predictors in this phase are confidence to overcome barriers, known as maintenance self-efficacy, and the formation of goal-directed plans, known as action planning.

While the HAPA has had demonstrable success in accounting for variance in intention toward, and actual participation in, health behaviors including PA [13], few studies have examined the utility of the HAPA in predicting PA participation in cancer survivors. One study has examined the applicability of the HAPA to increase PA in cancer survivors [14] and accounted for significant variance in intentions (49%) and PA (42%) in African-American breast cancer survivors [14]. However, this study predicted concurrent PA rather than prospective PA, which does not constitute a valid test of model predictions because the belief-based constructs in the theory explicitly refer to future rather than past or concurrent PA. It also did not examine the capacity of the model to account for a change in PA over time, which is critical to establish the predictive validity of the model. No study, to date, has examined the predictive validity of the HAPA in accounting for long-term PA change in cancer survivors. In addition, studies applying the HAPA have also tended to rely on self-report PA measures, which may increase prediction error because such measures are associated with reporting and recall bias [13]. The present study aims to address these shortcomings by adopting the HAPA to predict PA change in cancer survivors using non-self-report PA measures.

Our specific objective was to examine the utility of HAPA in predicting change in moderate-to-vigorous PA (MVPA) from baseline to 12 and 24 weeks among endometrial and colorectal cancer survivors. The study used data from the Wearable Activity Technology and Action Planning (WATAAP) trial, a randomized controlled trial (RCT) of a behavioral intervention aimed at promoting MVPA in cancer. Based on the HAPA, we predicted that changes in action self-efficacy, outcome expectancies, and risk perception would predict changes in intention. We also predicted that changes in action self-efficacy and maintenance self-efficacy would predict changes in action planning. In addition, we hypothesized that changes in intention would predict changes in action planning and PA behavior. Further, we predicted a direct effect of action planning change on PA change. Finally, changes in intention were hypothesized to predict changes in behavior mediated by changes in action planning.

## Method

### Study design

The data used in the current study were longitudinal data collected as part of the WATAAP trial, a RCT of a behavioral intervention aimed at promoting PA in endometrial and colorectal cancer survivors. The WATAAP intervention methods and protocol have been described previously [15, 16]. Participants completed self-report measures of the HAPA constructs (risk perception, action self-efficacy, maintenance self-efficacy, outcome expectancies, intention, and action planning) and PA behavior at baseline (T1), at 12 (T2), and 24 weeks (T3) post-intervention. The study was approved by the St. John of God Healthcare Human Research Ethics Committee (#1102) and registered with the Australia and New Zealand Clinical Trials Registry (Trial registration number ANZCTR2617000131358). Written informed consent was obtained from participants prior to enrolment. Eligibility criteria have been reported previously [15].

### Participant recruitment

Participant recruitment and eligibility criteria and the process of randomization have been reported elsewhere [15, 16]. In brief, participants were colorectal or gynecologic survivors (stages 1 and 2) with CVD risk factors, who had completed treatment in the previous five years, were in remission and insufficiently active (i.e., accumulating < 150 min of MVPA/week). Eligible participants were mailed letters from their treating oncologist inviting them to participate in the study. Colorectal and endometrial cancer survivors (*n* = 68, *M* age = 64.1 years, SD = 7.9) were randomized to intervention (*n* = 34) and control (*n* = 34) groups. Four participants dropped out of the trial in the first 12 weeks due to cancer recurrence (*n* = 1), unwillingness to be allocated to the control group (*n* = 1), lack of time (*n* = 1), and unwillingness to wear a Fitbit (*n* = 1). Baseline characteristics are published elsewhere [15].

### Data collection

Data were collected at each time point at St. John of God Subiaco Hospital by hospital staff blinded to group allocation. Participants were given a pen and paper questionnaire to complete covering demographic information and items based on the HAPA. Participants were also given an ActiGraph GT9X Link accelerometer, waistband, log, and postage materials and were instructed to wear the accelerometer for 7 consecutive days beginning the day following their assessment before mailing it back to the research team.

#### Moderate-to-vigorous physical activity

The ActiGraph GT9X Link was used to assess our primary outcome: time spent in MVPA in minutes/week. Participants wore the accelerometer on their right hip for all waking hours. Wear time had to exceed 10 h per day and contain no excessive counts (> 20,000) to be considered valid, with non-wear time defined as at least 60 consecutive minutes of zero counts. Data were processed using 60-s epochs. Daily accelerometer logs were completed by participants to allow for cross-checking of data. To estimate the mean minutes of MVPA per day, we used uniaxial Freedson cut points (≥ 1952 counts per minute) [17].

#### Health action process approach variables

Self-report measures of the HAPA variables were drawn from previously-published, validated items with Cronbach’s alpha scores for each ranging from 0.73 to 0.87 [18]. Items were amended to reference specific barriers identified by the sample [19–21]. Responses were provided on scales with 6-point response options. Full study measures are available in Appendix A (supplemental materials) and online: https://osf.io/6gu3r/?view_only=9e48e3625e084ca3a7526ea8b3eeea1b.

Risk perception was assessed through four items, based on a previous scale [22]. Items are scored on a six-point scale. Items measured perceived risk, vulnerability, likelihood, and a chance of developing health problems related to an inactive lifestyle (e.g., “I think it is likely that I will develop health problems related to obesity at some point in my life”).

Outcome expectations were assessed using twelve items. Five items were derived from the validated exercise pros subscale [23] and a further seven items were tailored to the present study based on formative research with cancer survivors [20, 21]. Items measured the extent to which participants agreed that regular PA over the next 12 weeks would help them attain key outcomes (e.g., “reduce tension or stress”; “feel more confident about my health”; “sleep better”; “have a positive outlook”; and “control my weight”).

Action self-efficacy was assessed through four items, based on previous research with breast cancer survivors [24]. Items assessed participants’ confidence to complete 150 min of MVPA/week (e.g., “I am confident I can do 150 min of moderate-intensity PA per week for the next 12 weeks”).

Maintenance self-efficacy was assessed using six items based on formative research on salient exercise barriers [20, 21]. Items measured confidence to participate in regular PA over the next 12 weeks in the face of salient barriers (e.g., “the weather is bad”; “I do not enjoy exercising”; “I do not have someone to encourage me to exercise”; “I am in a bad mood or feeling depressed”; “I can’t notice any improvements in physical fitness”; and “I can’t notice any improvements in my body”).

Action planning was assessed using four items based on an amended HAPA scale [25]. Participants were asked whether they had made a plan concerning what, when, where, and how they would engage in regular PA in the following 3 weeks.

Intention to participate in moderate-intensity PA was assessed using two items based on previous measures [26] (e.g., “I intend to participate in moderate-intensity PA for at least 150 min per week in the next 12 weeks”).

## Data analysis

In order to model change in the constructs of the constructs from the proposed HAPA model, we computed residualized change scores for each and used these scores to estimate the fit and pattern of effects in the proposed model using structural equation modeling. Each change score was computed by regressing the final follow-up (T2) score for each variable on its initial follow-up (T1) and baseline (T0) scores and extracting the unstandardized residual. The change score for each variable reflected the extent to which the variable changed in the variable from baseline. Change scores were computed using linear multiple regression. The education and income variables were dichotomized for the purpose of the main analyses to ease interpretation. Education was coded as lower (completed high or secondary school only) and higher (completed at least post-school training or qualification) education, and income was coded as lower (annual income Australian $52,000 or below) and higher (annual income Australian $52,001 or higher) income. These variables, along with age and gender, were included as covariates in each model in the main analysis.

**Fig. 1:**
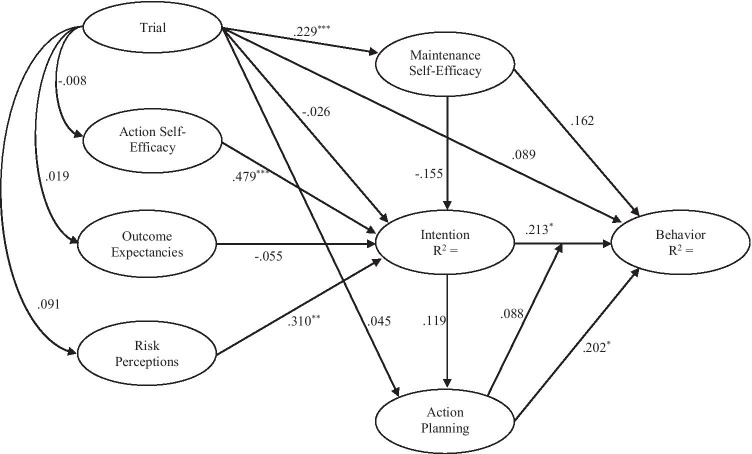
Proposed model based on the Health Action Process Approach (HAPA) predicting MVPA among cancer survivors including path coefficients from partial least squares structural equation modeling

The residualized change scores for each variable were used to estimate the proposed model based on the HAPA presented in Fig. 1. The model was estimated using variance-based structural equation modeling using the WARP 7.0 software [27]. The variance-based approach differs from the traditional covariance-based approach as it does not assume the normality of the data and is considered suitable for estimating models in smaller samples. In the analysis, the residualized change score for each variable from the proposed model was set as an indicator of a latent variable, and proposed effects were set as free parameters in the model. As we were primarily interested in testing the efficacy of the proposed model in predicting study outcomes, we also controlled for intervention effects by including a coded variable representing the intervention condition as an additional predictor of each latent variable in the model.

Missing data were imputed using linear multiple regression imputation [27]. The adequacy of the proposed model was established using multiple goodness-of-fit and model quality indices. Overall model fit was evaluated using Tenenhaus et al.’s goodness-of-fit (GoF) index [28], with values of 0.100, 0.250, and 0.360 corresponding to small, medium, and large effect sizes, respectively. Additional indices of model goodness-of-fit are provided by the average block variance inflation factor for model parameters (AVIF) and the average full collinearity variance inflation factor (AFVIF), which should be equal to or lower than 3.3 for well-fitting models. Model quality was also indicated by the average path coefficient (APC) and average *R*^2^ (AR^2^) coefficients, both of which should be statistically significant. In addition, four other indices were adopted to evaluate model quality: the Simpson’s paradox ratio (SPR), *R*^2^ contribution ratio (R^2^CR), the statistical suppression ratio (SSR), and the nonlinear bivariate causality direction ratio (NLBCDR). The SPR should exceed 0.700 and ideally approach 1.000, the R2CR and SSR should exceed 0.900 and 0.700, respectively, and the NLBCDR should exceed 0.700 for high-quality models. Model effects were estimated using standardized path coefficients with confidence intervals and test statistics. Effect sizes (ESs) were estimated using Cohen’s *f*-square coefficient, which represents *R*^2^ the contribution of each predictor variable to its respective dependent variable. Values of 0.02, 0.15, and 0.35 represent small, medium, and large ESs, respectively. Data files and analysis output are available online: https://osf.io/6gu3r/?view_only=9e48e3625e084ca3a7526ea8b3eeea1b

## Results

### Participants

Four participants were lost to follow-up resulting in a final sample of 64 participants (*M* age = 64.31; *SD* = 7.70; retention rate 94.12%). Full sample characteristics are presented in Appendix B (supplemental materials) and online: https://osf.io/6gu3r/?view_only=9e48e3625e084ca3a7526ea8b3eeea1b. The majority were colorectal cancer survivors (78%). A substantial proportion (36%) had a gross annual household income of Australian $52,000. Educational attainment was diverse with over half having either high school or post-school training and a further 48% achieving a university degree.

## Structural equation model

The proposed model (APC = 0.143, *p* = 0.054; AR^2^ = 0.087, *p* = 0.114; AVIF = 1.120; AFVIF = 1.265; GoF = 0.296; SPR = 0.938; R^2^CR = 0.980; SSR = 1.000; NLBCDR = 0.781) exhibited adequate model fit and quality indices according to the multiple criteria adopted. Standardized parameter estimates for the proposed direct effects for the model are summarized in Fig. 1 and Table 1, with full results reported in Appendix C (supplemental materials).

**Table 1:** Standardized path coefficients and effect sizes for direct and indirect effects for the partial least squares structural equation model of the proposed model based on the health action process approach

Effect	Β	ES
Direct effects		
Intention → MVPA	0.213^*^	0.040
Maintenance self-efficacy → MVPA	0.162	0.025
Action planning → MVPA	0.202^*^	0.058
Trial → MVPA	0.089	0.010
Action planning × intention → MVPA	0.088	0.020
Outcome expectancies → intention	− 0.055	0.013
Action self-efficacy → intention	0.479^***^	0.253
Risk perception → intention	0.310^**^	0.098
Maintenance self-efficacy → intention	− 0.155	0.042
Intention → action planning	0.119	0.014
Indirect effects		
Intention → action planning → MVPA	0.024	0.004
Outcome expectancies → intention → MVPA	− 0.012	< 0.001
Action self-efficacy → intention → MVPA	0.102	0.021
Risk perception → intention → MVPA	0.066	0.014
Maintenance self-efficacy → intention → MVPA	− 0.033	0.005
Total effects^a^		
Intention → MVPA	0.237^*^	0.044

^a^Total effect comprises the sum of indirect effects and the direct effect; *β* = standardized parameter estimate; SE = standard error; ES = Cohen’s *f*^2^ effect size

^*^p < 0.05^**^; *p* < 0.01; ^***^p < 0.001

Consistent with predictions, action self-efficacy (*β* = 0.479, *p* < 0.001, ES = 0.253) and risk perceptions (*β* = 0.310, *p* = 0.003, ES = 0.098) were statistically significant predictors of intentions, although effects of outcome expectancies on intention were small and not statistically significant (*β* =  − 0.055, *p* = 0.322, ES = 0.013). Similarly, consistent with predictions, intention (*β* = 0.213, *p* = 0.031, ES = 0.040) and action planning (β = 0.202, *p* = 0.039, ES = 0.058) significantly predicted MVPA. The effect of maintenance self-efficacy (*β* = 0.162, *p* = 0.212, ES = 0.025) was not statistically significant. Examination of the total effects of intention on MVPA revealed statistically significant effects (*β* = 0.237, *p* = 0.019, ES = 0.044). This is because there were small indirect effects of intentions on MVPA mediated by action planning, which although too small to be statistically significant in their own right, when combined with the direct effects contributed to the statistically significant total effects. Finally, effects of the intervention trial were not statistically significant, with the exception of the effect on maintenance self-efficacy (*β* = 0.229, *p* = 0.022, ES = 0.053). Overall, the model accounted for significant variance in intention (*R*^2^ = 0.381) and MVPA (*R*^2^ = 0.153).

## Discussion

This study examined the utility of the HAPA in predicting PA behavior change in survivors using non-self-report measures of PA using a prospective longitudinal design. Consistent with predictions, action self-efficacy and risk perceptions were statistically significant predictors of intentions. Intention and action planning were statistically significant predictors of MVPA. These findings are broadly consistent with meta-analytic research on the psychosocial predictors of PA in cancer survivors in which attitudes and self-efficacy were the strongest predictors of intentions, and intentions and self-efficacy were salient predictors of behavior [8]. Similarly, a meta-analysis of the HAPA in health behavior found prominent roles for action self-efficacy and risk perceptions in the prediction of intention; although, the effects were much smaller for risk perceptions (*β* = 0.066), intention, action planning, and maintenance self-efficacy as predictors of behavior [13].

An important finding in the present study was the larger effect of risk perceptions on intentions compared with effect sizes found in previous research [13, 29, 30]. The only other study examining the utility of the HAPA in cancer survivors found that severity did not predict intention or behavior [14]. A possible reason for these differences may be due to variation in the definition of constructs and measurement such as failing to link ill health with the specific health behavior. In the current study, we explicitly linked risk with experience health problems due to an inactive lifestyle, and this correspondence may have strengthened the effect on intention. Another explanation could be that physical inactivity is not widely viewed as a cardiovascular risk factor in general population studies. Findings from the present study suggest that risk perceptions are likely more pertinent in clinical populations and may have greater sensitivity to the link between physical inactivity and health risks. Research on the role of attitudes reinforces this interpretation: Hirschey et al. [8] found that instrumental and affective attitudes predicted PA in cancer survivors. Such findings contrast with meta-analyses that reveal affective attitudes as a better predictor of behavior in the general population [31, 32]. Recent work in cancer survivors also found instrumental attitude, which is closely linked with risk perceptions but not affective attitude predicted PA intention [33]. The perceived health benefits of PA encompassed by instrumental attitudes, similar to risk perceptions, may be more important in intention formation in clinical populations compared to nonclinical counterparts.

Consistent with the tenets of the HAPA, there were direct effects of action planning on PA. This aligns with meta-analytic research applying the HAPA to health behaviors and the only previous study applying the model to predict PA behavior in cancer survivors [13, 14]. The extent to which individuals report forming action plans to perform PA appears to be an important correlate of PA participation. This seems to be the case when behavior is measured concurrently with the HAPA constructs as reported previously [14], and, most importantly for PA behavior change, as reported in the current study. This may signal that capacity to plan may be an important self-enacted strategy for behavior change but also a potential technique that might be prompted in interventions [11].

Consistent with the HAPA [8, 13, 31] intention was a predictor of MVPA with a small-to-medium effect size. This modest association suggests that some participants followed through on their intentions, while others did not, as reported elsewhere [34]. This highlights an intention-behavior “gap” in the current sample. Although action planning was also included to address this “gap,” it did not mediate or moderate the relationship. This suggests that a critical hypothesis of the HAPA is not supported in the current sample, contrary to findings elsewhere [13]. However, these effects were confined to nonexperimental studies—intervention studies examining the moderating effect of planning in PA contexts have shown more promise [35] and are an avenue for further research in cancer survivors.

Although the effect of maintenance self-efficacy on MVPA in the current study was nonsignificant, the effect size was not trivial and approached statistical significance (*p* = 0.081). Maintenance self-efficacy is a consistent predictor of PA in previous research applying the HAPA [13, 30, 36]. This means that it would be premature to conclude a lack of an effect for maintenance self-efficacy on MVPA from the current data. Taking this into account, current findings are in line with other research applying the model in health behaviors. Further, one of the few studies that have examined this effect using non-self-report PA measures found that barrier management, a construct conceptually similar to maintenance self-efficacy, was associated with increased PA [37]. Taken together, findings suggest stage-specific self-efficacy may be relevant as a predictor of MVPA change in cancer survivors.

The model in the present study accounted for 15.3% of the variance in objective MVPA. While this is slightly lower than the range of variance explained (17–32%) in previous studies using the HAPA to predict PA [38–40], it is consistent with others applying the model in the health behavior domain. For example, Barg et al. [30] accounted for 15% of the variance in PA, and meta-analytic research has indicated that the model accounts for 17.5% of the variance in health behavior [13]. One possible source of variance may be the adoption of a non-self-report MVPA measure. Self-report measures introduce additional error variance when predicting relationships due to reporting and recall bias. We look to future research conducted in similar samples and using non-self-report MVPA measures to corroborate current findings.

## Study limitations

The present study had several strengths. There is a dearth of research that has examined the utility of the HAPA in examining PA change in cancer survivors. The longitudinal design of the study enabled the assessment of behavior change in the predictive relationships rather than examining “static” associations between constructs, a limitation of many other studies applying the model [13]. In addition, non-self-report measures of PA have rarely been used in the contexts of testing predictions of the HAPA, so our use of objectively measured MVPA represents another strength of the study. It is important, however, to acknowledge the limitations of the present study. First, the study did not comprehensively test the HAPA. Measures of coping planning, recovery self-efficacy, and action control were omitted for parsimony and to reduce participant response burden. Second, the sample size was relatively small, and findings may therefore not generalize to the wider population of cancer survivors. Finally, while the modeling of change enabled us to consider the temporal ordering of effects in the model, the current data are still correlational, so changes in model variables may still have been caused by effects of other, unmeasured extraneous variables. This precludes inference of causal links, and directional relations are therefore based on theory, not the data.

## Clinical implications

The present findings suggest the need to include behavior change techniques that strengthen intentions, foster self-efficacy, highlight risks associated with physical inactivity, and prompt action planning in interventions to promote PA in cancer survivors. For clinicians, the first step in promoting PA in patients is to prompt intention formation through highlighting the health risks associated with physical inactivity, the benefits of increased PA (i.e., raising the importance of exercise), and fostering confidence or self-efficacy to increase PA. The optimal way to foster confidence is through successful PA experiences achieved through effective goal setting and action planning. The development of a detailed plan of action including what (exercise dose, intensity, and duration), where (location of exercise), and when (day(s) and times) may assist in translating the intention into PA behavior change. However, it would be premature to base recommendations exclusively on the current data. Rather, these findings should contribute to the development of an evidence base to identify appropriate techniques to promote MVPA in cancer survivors.

## Conclusion

The current study provides preliminary support for key tenets of the HAPA in the context of PA change in cancer survivors. Intention and action planning change was a predictor of MVPA change. Further, change in action self-efficacy and risk perceptions predicted intention to change. The current findings provide some preliminary formative data that may inform the development of interventions aimed at promoting PA in cancer survivors.

## Supplementary Information

Below is the link to the electronic supplementary material.

Supplementary file1 (DOCX 86 KB)

## Data Availability

Data and analysis output are available online: https://osf.io/6gu3r/?view_only=9e48e3625e084ca3a7526ea8b3eeea1b.

## Code availability

Coding is available online: https://osf.io/6gu3r/?view_only=9e48e3625e084ca3a7526ea8b3eeea1b.
