# Supplementary material for: Predicting physical activity change in cancer survivors: an application of the Health Action Process Approach
Source: J Cancer Surviv. 2021 Sep 13;16(6):1176–83. doi: 10.1007/s11764-021-01107-6 (PMC9630182; doi:10.1007/s11764-021-01107-6)
Supplement: Supplementary file 1 — Supplementary file1 (DOCX 86 KB) [file 11764_2021_1107_MOESM1_ESM.docx]

Appendix A

*Items and Response Scales for the Health Action Process Approach Constructs*

| Variable | Item(s)/measure | Scale |
| --- | --- | --- |
| Outcome expectancies | For me, doing regular physical activity over the next 12-weeks will help me to…  …reduce tension or stress.  …feel more confident about one’s health.  …sleep better.  …have a positive outlook.  …control my weight.  …regain lost strength.  …prevent cancer recurrence.  …increase fatigue.  …increase joint pain.  …weaken my immune system.  …feel better about my body.  …increase my longevity. | 1 = disagree very strongly, 6 = agree very strongly |
| Risk perceptions | I think it is likely that I will develop health problems related to an inactive lifestyle at some point in my life.  Personally, I feel vulnerable to developing health problems related to an inactive lifestyle at some point in my life.  How likely do you think it is that you will get health problems related to an inactive lifestyle at some point in the future?  Compared to the average person, I feel that my chance of developing health problems related to an inactive lifestyle is… | 1 = disagree very strongly, 6 = agree very strongly  1 = disagree very strongly, 6 = agree very strongly  1 = extremely unlikely, 6 = extremely likely  1 = very much lower, 6 = very much higher |
| Action self-efficacy | I believe I have the ability to participate in moderate-intensity physical activity for at least 150-minutes per week over the next 12-weeks.  I am confident I can do 150-minutes of moderate-intensity physical activity per week for the next 12-weeks.  If I wanted to I could participate in 150-minutes of moderate-intensity physical activity per week for the next 12-weeks.  For me to do moderate-intensity physical activity for at least 150-minutes per week in the next 12-weeks would be... | 1 = disagree very strongly, 6 = agree very strongly  1 = extremely unconfident, 6 = extremely confident  1 = disagree very strongly, 6 = agree very strongly  1 = extremely difficult, 6 = extremely easy |
| Maintenance self-efficacy | I am confident I can participate in regular physical activity over the next 12-weeks when…  …the weather is bad.  …I.do not enjoy exercising.  …I do not have someone to encourage me to exercise.  …I am in a bad mood of feeling depressed.  …I can’t notice any improvements in fitness.  …I can’t notice any improvements in my body. | 1 = disagree very strongly, 6 = agree very strongly |
| Action planning | I have made a plan concerning ‘when’ I am going to engage in regular physical activity over the next three-weeks.  I have made a plan concerning ‘where’ I am going to engage in regular physical activity over the next three-weeks.  I have made a plan concerning ‘what’ kind of regular physical activity I will engage in over the next three-weeks.  I have made a plan concerning ‘how’ I am going to get to a place to engage in regular physical activity over the next three-weeks. | 1 = disagree very strongly, 6 = agree very strongly |
| Intention | I intend to participate in moderate-intensity physical activity for at least 150-minutes per week in the next 12-weeks.  I will try to participate in moderate-intensity physical activity for at least 150-minutes per week in the next 12-weeks. | 1 = disagree very strongly, 6 = agree very strongly |

Appendix B

*Sample Characteristics*

| Variable | Value |
| --- | --- |
| Participants (final sample) | 64 |
| Age, *M* years (SD) | 54.31 (7.70) |
| Gender, *n* (%)^a^ |  |
| Female | 32 (50.0) |
| Male | 32 (50.0) |
| Marital status, *n* (%)^c^ |  |
| Married | 48 (75.0) |
| In a relationship | 2 (3.1) |
| Single | 6 (9.4) |
| Separated/divorced | 7 (10.9) |
| Widowed | 1 (1.6) |
| Ethnicity, *n* (%)^d^ |  |
| Caucasian/White | 62 (96.9) |
| South Asian (Indian) | 2 (3.1) |
| Income, *n* (%)^e^ |  |
| Under $30,000 | 6 (9.4) |
| $30,001 to $52,000 | 17 (26.6) |
| $52,001 to $104,000 | 20 (31.3) |
| $104,001 to $156,000 | 11 (17.2) |
| $156,001 + | 9 (14.1) |
| Missing | 1 (1.6) |
|  |  |
| Education level, *n* (%) |  |
| University degree | 31 (48.4) |
| Post-school training/qualification | 15 (23.4) |
| Completed high/secondary school | 18 (28.1) |
| Cancer diagnosis |  |
| Colorectal | 50 (78.1) |
| Endometrial | 13 (20.3) |
| Colorectal and endometrial | 1 (1.6) |
| Chemotherapy treatment |  |
| Yes | 29 (45.3) |
| No | 35 (54.7) |
| Radiotherapy treatment |  |
| Yes | 15 (23.4) |
| No | 49 (76.6) |
| Brachytherapy treatment |  |
| Yes | 3 (4.7) |
| No | 61 (95.3) |
| Hormone therapy |  |
| Yes | 1 (1.6) |
| No | 63 (98.4) |
| Cardiovascular disease diagnosis |  |
| Yes | 6 (9.4) |
| No | 58 (90.6) |

Appendix C

*Full Results of the Partial Least Squares Structural Equation Model of the Proposed Model Based on the Health Action Process Approach*

| Effect | Β | *p* | SE | ES |
| --- | --- | --- | --- | --- |
| Direct effects |  |  |  |  |
| Intention→MVPA | .213 | .031 | .112 | .040 |
| Maintenance self-efficacy→MVPA | .162 | .081 | .114 | .025 |
| Action planning→MVPA | .202 | .039 | .113 | .058 |
| Trial→MVPA | .089 | .225 | .117 | .010 |
| Action planning x Intention→MVPA | .088 | .228 | .117 | .020 |
| Outcome expectancies→Intention | -.055 | .322 | .118 | .013 |
| Action self-efficacy→Intention | .479 | <.001 | .103 | .253 |
| Risk perception→Intention | .310 | .003 | .109 | .098 |
| Maintenance self-efficacy→Intention | -.155 | .089 | .114 | .042 |
| Intention→Action planning | .119 | .155 | .116 | .014 |
| Trial→Intention | -.026 | .413 | .119 | .001 |
| Trial→Outcome expectancies | .019 | .438 | .120 | <.001 |
| Trial→Action self-efficacy | -.008 | .475 | .120 | <.001 |
| Trial→Risk perception | .091 | .220 | .117 | .008 |
| Trial→Maintenance self-efficacy | .229 | .022 | .112 | .053 |
| Trial→Action planning | .045 | .352 | .119 | .002 |
|  |  |  |  |  |
| Indirect effects |  |  |  |  |
| Intention→Action planning→MVPA | .024 | .389 | .084 | .004 |
| Outcome expectancies→Intention→MVPA | -.012 | .445 | .085 | <.001 |
| Action self-efficacy→Intention→MVPA | .102 | .110 | .082 | .021 |
| Risk perception→Intention→MVPA | .066 | .215 | .083 | .014 |
| Maintenance self-efficacy→Intention→MVPA | -.033 | .348 | .084 | .005 |
|  |  |  |  |  |
| Total effects^a^ |  |  |  |  |
| Intention→MVPA | .237 | .019 | .111 | .044 |

*Note*. ^a^Total effect comprises the sum of all indirect effects and the direct effect; β = Standardized parameter estimate; SE = Standard error; ES = Cohen’s *f*^2^ effect size; Trial = Contrast code for intervention condition.
